# Comparison of Early and Late Surgeries after Coronary Stent Implantation in Patients with Normal Preoperative Troponin Level: A Retrospective Study

**DOI:** 10.3390/jcm12072524

**Published:** 2023-03-27

**Authors:** Sang Hyun Lee, Eun Kyung Lee, Hyun Joo Ahn, Sangmin M. Lee, Jie Ae Kim, Mikyung Yang, Ji Won Choi, Jeayoun Kim, Heejoon Jeong, Seungmo Kim, Jinseo Kim, Joonghyun Ahn

**Affiliations:** 1 Department of Anesthesiology and Pain Medicine, Samsung Medical Center, Sungkyunkwan University School of Medicine, Seoul 06351, Republic of Korea; 2 Biomedical Statistics Center, Data Science Research Institute, Research Institute for Future Medicine, Samsung Medical Center, Seoul 06351, Republic of Korea

**Keywords:** coronary stents, noncardiac surgery, troponin I, stent to surgery time interval

## Abstract

Current guidelines recommend delaying noncardiac surgery for 6 months after drug eluting stent implantation. However, this recommendation is largely based on limited evidence and various event definitions. Whether early surgery within 6 months of coronary stent implantation increases myocardial injury in patients with normal preoperative high-sensitivity cardiac troponin I (hs-cTnI) has not yet been investigated. This retrospective study assessed patients who received coronary stent implantation and underwent noncardiac surgery (vascular, abdominal, or thoracic) between 2010 and 2017 with normal preoperative hs-cTnI (n = 186). Patients were divided into early (within 6 months of PCI) and late (after 6 months of PCI) groups. The primary endpoint was the incidence of myocardial injury as diagnosed by hs-cTnI within 3 days post-operation. The secondary outcomes were myocardial infarction, stent thrombosis, emergent coronary revascularization, major bleeding (bleeding requiring transfusion or intracranial bleeding), stroke, renal failure, heart failure, or death within 30 days post-operation. Inverse probability treatment weighting (IPTW) was carried out to adjust for the intergroup baseline differences. Myocardial injury occurred in 28.6% (8/28) and 27.8% (44/158) of the early and late groups, respectively, with no difference between groups (odds ratio [OR] 1.067, 95% confidence interval [CI] 0.404, 2.482; *p* = 0.886). Secondary outcomes did not differ between the groups. IPTW analysis also showed no differences in myocardial injury and secondary outcomes between the groups. In conclusion, early surgery within 6 months after coronary stent implantation did not increase the incidence of myocardial injury in patients with normal preoperative hs-cTnI.

## 1. Introduction

Now that percutaneous coronary interventions (PCI) are performed worldwide, the incidence of noncardiac surgery (NCS) after coronary stent implantation is 10 to 20% within the second year of stent insertion [1,2].

The proper time interval from “coronary stent to surgery” has been the subject of debate. The interval between “stent to surgery” is determined by risk-benefits that consider the possibility of stent thrombosis associated with premature cessation of antiplatelets, bleeding associated with the continuous use of antiplatelets, and adverse patient outcomes from delayed surgery. Current practice guidelines of the American Society of Anesthesiologists recommend that elective noncardiac surgery be postponed for at least 12 months until endothelization of the drug eluting stent (DES) is completed [3,4]. The 2016 American College of Cardiology and the American Heart Association (ACC/AHA) recommends waiting 6 months in cases of stable coronary artery disease (class of recommendation: 1, level of evidence: B-non-randomized) [5]. With advances in coronary stent techniques and improved patient care, a shorter interval of “stent to surgery” is increasingly recommended by more recent guidelines [5,6,7]. However, some anesthesiologists may choose to practice more conservatively as these guidelines are based on limited and weak evidences drawn from non-randomized trials [1,8,9,10,11,12]. 

In previous studies, patients who underwent surgery after PCI were compared to those who received coronary stents but did not undergo surgery [12], or patients who underwent surgery but did not have coronary stents [13,14,15]. Few studies directly compared those who underwent early or late surgery. In addition, previous studies focused on composite complications of major adverse cardiac events (MACEs) such as myocardial infarction (MI), bleeding, stroke, or death to determine the appropriate timing for noncardiac surgery after PCI [1,2,8,13,16,17,18,19,20,21,22], rather than relying on a universal direct marker of cardiac injury. 

High-sensitivity cardiac troponin I (hs-cTnI) is used as an objective early marker for postoperative myocardial injury. To the best of our knowledge, there were no studies that compared changes in hs-cTnI between early and late surgeries after PCI, and no information is available on postoperative outcomes in patients who had normal hs-cTnI before surgery. 

In this single-center retrospective study, we evaluated whether early surgery (within 6 months of PCI) increases the incidence of myocardial injury, as diagnosed by hs-cTnI level, compared with late surgery (after 6 months of PCI) in coronary stented patients who underwent vascular, abdominal or thoracic surgery with normal preoperative hs-cTnI. Inverse probability treatment weighting (IPTW) was performed to compare early and late groups, as few patients undergo surgery within 6 months of PCI. 

## 2. Materials and Methods

### 2.1. Patients

All patients (n = 2517) who received PCI and underwent noncardiac surgery (vascular, abdominal or thoracic surgery) between January 2010 and March 2017 at Samsung Medical Center, Seoul, Republic of Korea were assessed. Only the first index surgery within 30 days of a single admission was included. The stent to surgery interval was based on the most recent coronary stent implantation before the surgery if the patient had more than one PCI episode. Patients were divided into early (within 6 months of PCI) or late (after 6 months of PCI) groups.

### 2.2. Inclusion and Exclusion Criteria

**Figure 1:**
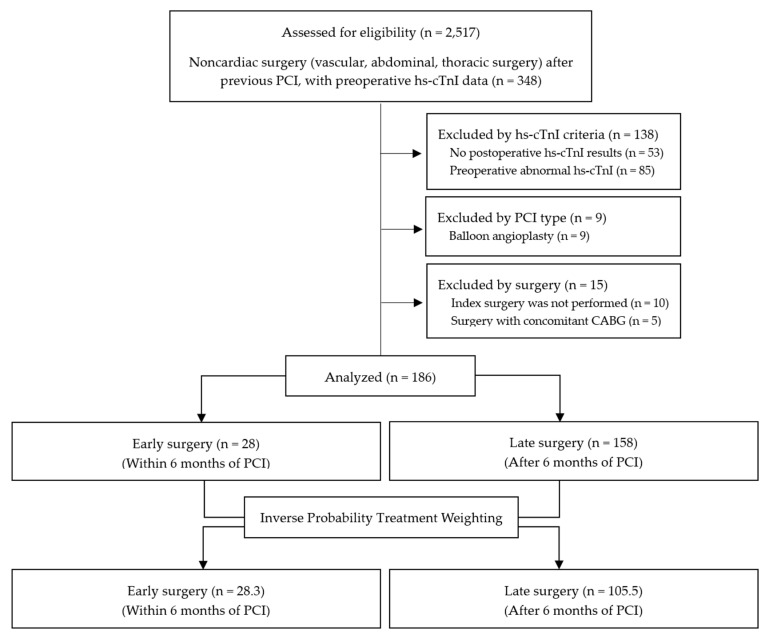
Flow diagram. PCI, percutaneous coronary intervention; Hs-cTnI, high-sensitivity cardiac troponin I; CABG, coronary artery bypass surgery.

Coronary stented patients with pre- and post-operative hs-cTnI results undergoing the index surgery were included. The exclusion criteria were as follows: (1) no preoperative hs-cTnI test, (2) abnormal preoperative hs-cTnI level, (3) no postoperative hs-cTnI test within 3 days post-operation, (4) previous PCI with balloon angioplasty without stent implantation, (5) operation other than the index surgery, and (6) concomitant coronary artery graft surgery (Figure 1).

### 2.3. Data Acquisition

Data were collected using our hospital’s electronic medical records. Reviewed data include patient characteristics; underlying disease; echocardiographic findings; revised cardiac risk index (RCRI); laboratory data including preoperative N-Terminal-proB-type Natriuretic Peptide (NT-proBNP); surgery type; emergency surgery; estimated blood loss; intraoperative transfusion; surgery duration; cause of coronary stent implantation, coronary stent type, number, and site(s); and discontinued days of antiplatelets before surgery.

### 2.4. Outcomes and Follow-Up

The primary outcome was myocardial injury, assessed by hs-cTnI within 3 days post- operation. The secondary outcomes were myocardial infarction, stent thrombosis, and the need for emergent coronary revascularization, as well as bleeding requiring transfusion or intracranial bleeding within 30 days post-operation. Other complications such as stroke, renal failure, heart failure, or death within 30 days post-operation were collected. The postoperative duration of the hospital stay was also obtained.

Myocardial injury was defined as any hs-cTnI result exceeding 0.04 ng/mL within 3 days of the operation. The lower detection limit was 0.006 ng/mL, and the normal range was ≤0.04 ng/mL according to the 99th percentile reference. Levels were measured using a highly sensitive immunoassay with an automated analyzer (Advia Centaur XP, Siemens Healthcare Diagnostics, Erlangen, Germany). Myocardial infarction was assessed using the third universal definition [23], which is a cardiac biomarker (hs-cTnI) elevation of at least one value above the 99th percentile upper reference limit, in addition to one of the following criteria: symptoms, cardiac echocardiographic diagnosis, or electrocardiogram change indicating myocardial ischemia. Major bleeding was defined as significant bleeding requiring transfusion or an intracranial hemorrhage. Stroke was diagnosed by neurologic symptoms and brain magnetic resonance imaging. Heart failure was defined as dyspnea, pulmonary congestion, and elevated NT-proBNP, or as noted on echocardiography.

### 2.5. Statistical Analysis

For the comparison of early and late groups in baseline patient characteristics and outcome data, continuous variables were analyzed using the Student’s *t*-test or the Mann–Whitney *U* test, according to the normality of data as evaluated with the Shapiro–Wilk test. Data are presented as mean (standard deviation) or median (interquartile range) as appropriate. Categorical variables were analyzed by Chi-square test or Fisher’s exact test as appropriate and are described as number (%).

### 2.6. IPTW

The “stent to surgery” interval may have been affected by several patient characteristics, and the early and late group may show differences in baseline demographics. Thus, IPTW was performed to adjust for these intergroup differences in age, sex, body mass index (BMI), American Society of Anesthesiologist Physical Status (ASA PS; II, III vs. IV), surgery type (major vascular vs. non major vascular), emergency surgery, etiology of stent insertion (acute myocardial infarction vs. angina pectoris), preoperative antiplatelets (or anticoagulant) use, discontinued days (duration) of antiplatelets, and preoperative comorbidity including diabetes mellitus (DM), hypertension (HTN), chronic kidney diseases (CKD), atrial fibrillation, peripheral vascular diseases, and transient ischemic attack (TIA) or stroke. A weight of mean of propensity score (PS)/PS was assigned to the early group and (1-means of PS)/(1-PS) to the late group, with PS being the probability of being assigned to the early group. The ability of the model to balance the cohort characteristics in a pseudo-population was assessed using standardized mean differences. Simple logistic regression was performed for the primary endpoint, and double adjustments using the weighted multiple logistic regression were carried out to adjust for variables with standardized mean difference >0.1 even after IPTW (adjusted odds ratio and 95% confidence interval) [24].

To identify confounding factors for myocardial injury, multiple logistic regression was carried out, with a stepwise selection (likelihood ratio, enter if *p* < 0.05 and remove variable if *p* > 0.2) in the unweighted raw data. Variables assessed for the multiple logistic regression are as follows: the early group vs. the late group was set as a fixed variable, plus age, sex, BMI, surgery type, emergency surgery, ASA PS, etiology of stent insertion, DM, use of DM medications (metformin, sulfonylurea, dipeptidyl peptidase-4 inhibitor), HTN, CKD, atrial fibrillation, peripheral vascular disease, stroke including TIA, preoperative antiplatelets, and discontinuation of antiplatelets.

### 2.7. Sample Size Calculation

We performed a sample size justification for this retrospective analysis. Since there was no report on the incidence of myocardial injury diagnosed by hs-cTnI in coronary stented patients undergoing noncardiac surgery, we based it on the incidence of myocardial injury after noncardiac surgery, which ranged from 5 to 20% in the previous literature [25,26]. On the presumption that the incidence of myocardial injury in the late group was 10% compared to that of 35% in the early group, 25 patients in the early group and 100 patients in the late group would have the power of 83%.

All *p* values were two-sided, and *p* < 0.05 indicated a significant difference. Rex Excel-based statistical analysis software ver. 3.6.1 (RexSoft, Seoul, Republic of Korea, http://rexsoft.org/, accessed on 1 November 2022) based on R ver. 4.0.0 (R Foundation for Statistical Computing, Vienna, Austria) and IBM SPSS^®^ Statistics for Windows version 22.0 (IBM Corp., Armonk, NY, USA) were used to conduct all analyses.

## 3. Results

In this single-center retrospective study, all patients who underwent noncardiac surgery (vascular, abdominal, or thoracic surgery) after PCI between January 2010 and March 2017 were assessed (n = 2517). Among them, those with preoperative hs-cTnI results were selected (n = 348). Patients with no postoperative hs-cTnI results within 3 days post-operation (n = 53), or with high preoperative hs-cTnI results indicating ongoing cardiac injury (n = 85), or those who underwent balloon angioplasty only for PCI (n = 9), or those who did not receive the index surgery (n = 10), or those who received concomitant coronary artery bypass surgery (n = 5) were all excluded. Finally, 186 patients were analyzed for myocardial injury. Data on primary and secondary outcomes were available in all analyzed patients (Figure 1).

### 3.1. Demographics Data and Operative Characteristics

In the raw data, the baseline characteristics of patients in the early and late groups did not differ except for preoperative use of dual antiplatelet therapy (96% vs. 38%, *p* < 0.001), which was more common in the early group (Table 1). Most patients received clopidogrel and/or aspirin as antiplatelet therapy. The discontinued days of preoperative antiplatelets (or anticoagulant) were similar between the groups (3.4 vs. 4.4 days, early vs. late group; *p* = 0.25) (Table 1).

### 3.2. Myocardial Injury and Postoperative Complications

**Figure 2:**
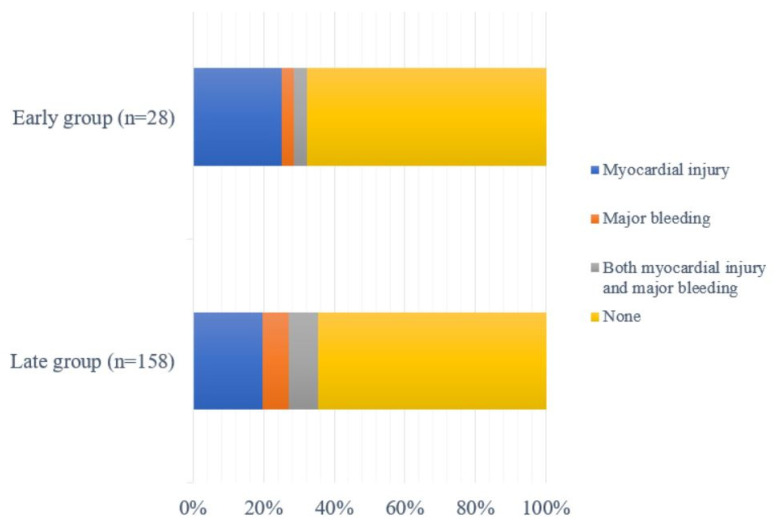
Incidence of composite events (myocardial injury or major bleeding) between early (9/28 [32.2%]) and late groups (56/158 [35.4%]) were not different (*p* = 0.690). Major bleeding included bleeding requiring transfusion or intracranial hemorrhage within 30 days post-operation.

In the raw data, the incidence of myocardial injury was 28.0% (52/186). There was no difference in myocardial injury between the early and late groups (28.6% [8/28] vs. 27.8% [44/158]; OR 1.067, 95% CI 0.404, 2.482; *p*= 0.886). Myocardial infarction occurred in nine patients, all in the late group (0% [0/28] vs. 5.7% [9/158]; OR 0.276, 95% CI 0.000, 2.276; *p* = 0.398) Among these patients with myocardial infarction, four showed changes in ST, two of whom required emergent coronary revascularization for non-ST segment elevation myocardial infarction (NSTEMI) (0% [0/28] vs. 1.3% [2/158]; OR 1.098, 95% CI 0.000, 13.648; *p* = 0.953). None of the patients experienced stent thrombosis or restenosis. The incidence of major bleeding was 14.5% (27/186), with no group difference (7.1% [2/28] vs. 15.8% [25/158]; OR 0.494, 95% CI 0.064, 1.645; *p* = 0.319) (Table 2). The composite incidence of either myocardial injury or major bleeding did not differ between the early and late groups (32.1% [9/28] vs. 35.4% [56/158]; *p* = 0.690) (Figure 2).

### 3.3. IPTW

The IPTW matched cohort is shown in Appendix A Table A1. After IPTW, a pseudo-population was created, and the incidence of myocardial injury was 31.1% (8.8/28.3) vs. 30.8% (32.5/105.5) of patients in the early vs. late group (OR 1.035, 95% CI 0.400, 2.447; *p* = 0.939) (Table 2). Double adjustment results for a standardized mean difference >0.1 after IPTW also showed no significant differences between the early and late groups in myocardial injury (OR 1.125, 95% CI 0.465, 2.723; *p* = 0.795). After IPTW, secondary outcomes did not differ between the groups (Table 2).

In the raw data, the differences in other major complications including heart failure, stroke, renal failure, Clavien–Dindo surgical complications, postoperative hospital stays, and in-hospital mortality were not significant statistically. One patient in the late group died due to cerebral infarction and subsequent heart failure (Table 2).

Table 3 shows the simple logistic regression and multiple logistic regression of raw data to identify contributing factors to myocardial injury. Early or late surgery was not associated with myocardial injury. Major vascular surgery was the only variable that increased the odds of myocardial injury significantly (adjusted OR 5.060, 95% CI 2.407, 10.635; *p* < 0.001).

## 4. Discussion

Using a definition of myocardial injury as elevated hs-cTnI, early noncardiac surgery within 6 months of coronary stent implantation did not increase the incidence of myocardial injury in patients with normal preoperative hs-cTnI. The incidence of major bleeding also did not differ between the groups.

In our study, the incidence of myocardial injury based on hs-cTnI was 28.6% and 27.8% in the early and late groups, respectively. This is higher than that of the Godet et al.’s study, where the incidence was 12% in 96 consecutive patients who underwent noncardiac surgery after PCI [27]. This difference may be attributable to our use of a high sensitivity troponin assay, hs-cTnI, which may be a more accurate marker for myocardial injury than the troponin used in Godet et al. [27].

Previous studies focused on a wide range of composite outcomes of MACEs. However, MACE may not be directly related to coronary stent in surgical population. Our one heart failure case was due primarily to massive bleeding from surgical complications and our mortality case was due to cerebral infraction and subsequent heart failure, neither of which was accompanied by elevated hs-cTnI. If the rationale for delaying surgery is to wait for stent endothelization and safe cessation of dual antiplatelet therapy, it appears to be more appropriate to focus on the measurement of hs-cTnI (primary outcome) and the occurrence of myocardial injury or infarction and stent thrombosis/revascularization (secondary outcomes) to determine “stent to surgery” time [5,27].

It is not clear why the rate of myocardial injuries did not differ between the two groups. First, this study included patients with normal preoperative hs-cTnI. According to previous studies, noncardiac surgery after acute coronary syndrome has a high risk of MACE or myocardial infarction, but stable coronary artery diseases have a low risk of MACE regardless of the timing of the surgery [5,10]. Second, another possible explanation involves the type 2 mechanism of myocardial injury in surgical patients [28]. During the perioperative period, myocardial supply-demand mismatch (type 2) is more common than stent thrombosis (type 1) [29,30], which may be treated conservatively and not require coronary artery intervention. In our study, no patients who manifested myocardial injury required coronary artery intervention, and all were treated conservatively, except for two patients in the late group, who had to be referred to cardiologists for emergency revascularization. If the type 2 mechanism is the major contributor of myocardial injury, delaying surgery would not reduce it. Third, coronary stent type may also have contributed to the lack of difference between the two groups. Recent generation DESs were designed using thinner stent platforms and thrombo-resistant, bioabsorbable, or biocompatible polymers [31]. These newer polymers minimize inflammation [32,33] and result in lower rates of stent thrombosis [33,34,35]. Stent thrombosis occurred in only 1.5% of patients over a 3-year follow up [36]. Emerging data suggest that it may be safe to discontinue dual antiplatelet therapy as early as 3 months after implantation of a new generation stent [33,34,35]. Because of the retrospective nature of the study, we were unable to identify the generation of stents used in some patients. However, the early surgery group received a new generation DES more frequently than did the late group, despite the missing data. Lastly, some antidiabetics such as metformin [37]_,_ glucagon-like peptide 1 (GLP-1; incretins) analogs [38,39], or SGLT2 (Sodium-glucose cotransporter 2) inhibitors [40,41] are known to have a favorable effect against myocardial infarction. Patients in our study were not receiving GLP-1 analogs and/or SGLT2 inhibitors, but some were taking DPP-4-inhibitors, which indirectly increase GLP-1. In our study, patients who were taking metformin (14% vs. 18%; early vs. late group) and DPP-4-inhibitor (11% and 13%; early vs. late group) were not different between the groups. These drugs may have affected the result of no difference in the rate of myocardial injury.

In our study, preoperative use of dual antiplatelet therapy was more common in the early group than the late group (96% vs. 38%, *p* < 0.001 in unweighted cohort data), but the duration of preoperative antiplatelet discontinuation was similar between the groups (3.4 days and 4.4 days; *p* = 0.25 in unweighted cohort data). The risk of stopping antiplatelet therapy is not consistent among previous studies. The RECO study found an increased incidence of MACE with a complete cessation of antiplatelets after noncardiac surgery [8], but other studies reported no differences between the stop and the continued use [2,9,42]. Some studies reported even higher incidences of MACE in patients with greater platelet inhibition [19,29] and those with continuous antiplatelet medication [20]. In our multiple logistic regression (Table 3), types and discontinued duration of antiplatelets were not contributing variables for the occurrence of myocardial injury. Antiplatelet therapy should be tailored to the patient depending on the cause of PCI, inherent surgical risk of bleeding, and type of stent [1,18,20], and the consensus decision should be based on the opinions of the cardiologist, surgeon, and anesthesiologist [5,6,7,43].

Our study has several limitations. First, the small number of patients in this study limits generalizability and makes it difficult to draw a concrete conclusion. However, inclusion of minor or outpatient surgery is likely to produce more favorable outcomes for early surgery [12]. A large cohort study is required in future. Second, the retrospective nature of this study has resulted in a lack of information on the type of coronary stent and the etiology of stent implantation in some patients. Third, our cohort is Asian. East-Asians are reportedly to show a decreased thrombotic risk compared to other ethnicities [44]. Fourth, as a retrospective study, perioperative management was not controlled, especially in relation to restarting antiplatelets or postoperative thromboprophylaxis. However, if clinically acceptable, antiplatelet therapy was routinely resumed on the first postoperative day as an institutional protocol.

## 5. Conclusions

In conclusion, early surgery within 6 months of coronary stent implantation may not increase the incidence of myocardial injury in patients with normal preoperative hs-cTnI. The decision on the timing of surgery may be tailored for each patient by the consensus of surgeons, cardiologists, and anesthesiologists rather than by a strict adherence to the guidelines.

## Figures and Tables

**Table 1:** Patient and operative characteristics between early and late surgery (raw data).

Variables	Early Surgery	Late Surgery	*p*
	(n = 28)	(n = 158)	
Age, year	67.3 (8.1)	68.3 (8.3)	0.55
Female	1 (4)	18 (12)	0.32
BMI, kg/m^2^	25.0 (2.6)	24.6 (3.2)	0.54
Weight, kg	68.7 (7.4)	67.9 (10.6)	0.71
Height, cm	165.8 (6.2)	165.9 (7.5)	0.91
ASA PS			0.06
II	7 (25)	74 (47)	
III	20 (71)	81 (51)	
IV	1 (4)	3 (2)	
Hypertension	18 (64)	113 (72)	0.44
Diabetes mellitus	11 (39)	62 (39)	>0.99
Metformin	4 (14)	28 (18)	
Sulfonylurea	2 (7)	21 (13)	
Dipeptidyl Peptidase-4 inhibitor	3 (11)	21 (13)	
Insulin	1 (4)	7 (4)	
Alpha glucosidase	0 (0)	2 (1)	
Thiazolidinediones	1 (4)	0 (0)	
No antidiabetic medication	2 (7)	5 (3)	
Stroke or transient ischemia attack	5 (18)	35 (22)	0.61
Chronic kidney disease	1 (4)	18 (11)	0.32
Structural heart disease _a_	3 (11)	21 (13)	>0.99
Left ventricular ejection fraction < 30%	0 (0)	2 (1)	>0.99
Atrial fibrillation	2 (7)	11 (7)	>0.99
Peripheral vascular disease	5 (18)	27 (17)	>0.99
Revised cardiac risk index			0.74
0	2 (7)	9 (6)	
1	14 (55)	91 (60)	
2	9 (32)	49 (31)	
3	3 (11)	9 (6)	
Serum creatinine ≥ 2.0 mg/dL	1 (4)	12 (8)	0.70
Hemoglobin, g/dL	12.5 (1.9)	12.7 (2.1)	0.62
CRP, mg/dL _b_	0.13 (0.06, 0.71)	0.25 (0.07,0.88)	0.58
NT-proBNP, ng/dL _c_	88.2 (46.6, 715.0)	148 (67.5, 503.4)	0.75
Albumin, g/dL	4.0 (0.5)	4.0 (0.5)	0.59
Cholesterol	123.9 (37.1)	134.8 (32.1)	0.11
LDL cholesterol _d_	66.5 (25.7)	75.3 (25.3)	0.16
Glucose, mg/dL	136.8 (46.3)	129.7 (53.9)	0.52
Surgery			
Major vascular	7 (25)	39 (25)	0.74
Non major-vascular (sub-category, below)	21 (75)	119 (75.3)	
Cholecystectomy	2 (7)	16 (10)	
Gastrectomy	5 (18)	10 (7)	
Hepatobiliary	1 (4)	17 (11)	
Colorectal surgery	2 (7)	10 (6)	
Nephrectomy-cystectomy	0 (0)	2 (1)	
Kidney transplantation	0 (0)	2 (1)	
Liver transplantation	0 (0)	5 (3)	
Other abdominal surgery	10 (36)	62 (39)	
Thoracic surgery	5 (18)	21 (13)	
Carotid endarterectomy	6 (21)	36 (23)	
Emergency surgery	3 (11)	13 (8)	0.71
Intraoperative estimated blood loss, ml	125 (80, 450)	200 (100, 700)	0.33
Intraoperative transfusion	4 (14)	43 (27)	0.15
Surgery duration, min	174.5 (117, 206)	192 (122, 262)	0.21
*Coronary stent data*			
Causes of coronary stent implantation			0.99
Acute Myocardial Injury	9 (32)	52 (33)	
Angina pectoris	17 (61)	94 (60)	
Not known	2 (7)	12 (7)	
Coronary stent type			0.11
DES	16 (57)	65 (41)	
First generation	1 (6)	26 (40)	
Durable polymer coated	11 (69)	29 (45)	
Biodegradable polymer coated	2 (13)	4 (6)	
Polymer free drug coated	1 (6)	0 (0)	
Unknown DES type	1 (6)	6 (9)	
BMS	4 (14)	14 (9)	
Unknown stent type	8 (29)	79 (50)	
Coronary stent number_b_			0.61
1	17 (63)	76 (67)	
2	7 (26)	30 (27)	
3	3 (11)	7 (6)	
Coronary stent site_b_			0.10
Left anterior descending	16 (57)	62 (39)	
Antiplatelets (or anticoagulants) use			<0.001
None	0 (0)	3 (2)	
Aspirin only	1 (4)	70 (44)	
Clopidogrel only	0 (0)	22 (14)	
Dual (aspirin + clopidogrel)	27 (96)	60 (38)	
Warfarin	0 (0)	3 (2)	
Discontinued days of any antiplatelets (or anticoagulants)	3.4 (3.1)	4.4 (4.0)	0.25

Values are presented as mean (standard deviation) or median (interquartile range) or number (%). Early vs. Late surgery: surgical time from coronary stent implantation within 6 months vs. after 6 months. BMI, body mass index; ASA PS, American Society of Anesthesiologist Physical Status; NT-proBNP, N-terminal pro hormone B-type natriuretic peptide N-terminal; CRP, C-reactive protein; LDL, low density lipoprotein; DES, drug eluting stent; BMS, bare metal stent._a_ Structural heart disease included regional wall motion abnormality or valvular heart disease. _b_ early surgery n = 28, late surgery n = 138. _c_ early surgery n = 20, late surgery n = 114. _d_ early surgery n = 20, late surgery n = 111.

**Table 2:** Myocardial injury and other complications between early and late surgery before and after IPTW.

Variables	Unmatched Cohort				Weighted Cohort after IPTW			
	Early Surgery (n = 28)	Late Surgery (n = 158)	Odds Ratio (95% CI) _a_	*p*	Early Surgery (n = 28.3)	Late Surgery (n = 105.5)	Odds Ratio (95% CI) _b_	*p*
Myocardial injury	8 (28.6)	44 (27.8)	1.067 (0.404, 2.482)	0.886	8.8 (31.1)	32.5 (30.8)	1.035 (0.400, 2.447)	0.939
Myocardial infarction	0 (0)	9 (5.7)	0.276 (0.000, 2.276)	0.398	0.0 (0.0)	6.9 (6.6)	0.232 (0.000, 1.995)	0.330
MACE	3 (10.7)	23 (14.6)	0.791 (0.159, 2.367)	0.707	3.3 (11.8)	17.4 (16.5)	0.748(0.163, 2.251)	0.636
In-stent thrombosis	0	0	NA	NA	0	0	NA	NA
Emergent coronary revascularization	0 (0)	2 (1.3)	1.098 (0.000, 13.648)	0.953	0.0 (0.0)	1.9 (1.8)	0.746 (0.000, 9.460)	0.854
Major bleeding	2 (7.1)	25 (15.8)	0.494 (0.064, 1.645)	0.319	0.8 (3.0)	18.1 (17.1)	0.227 (0.006, 1.014)	0.113
Stroke	0 (0)	3 (1.9)	0.779 (0.000, 8.248)	0.872	0.0 (0.0)	1.9 (1.8)	0.758 (0.000, 9.711)	0.862
Thrombosis	1 (3.6)	4 (2.5)	1.873 (0.072, 10.547)	0.522	0.8 (3.0)	2.8 (2.6)	1.516 (0.038, 10.540)	0.695
Heart failure	2 (7.1)	7 (4.4)	1.906 (0.239, 7.607)	0.403	2.5 (8.8)	5.6 (5.3)	1.892 (0.300, 7.697)	0.394
Newly onset atrial fibrillation or flutter	1 (3.6)	13 (8.2)	0.588 (0.022, 2.566)	0.555	0.8 (3.0)	10.5 (10.0)	0.416 (0.010, 1.973)	0.358
New dialysis	0 (0)	3 (1.9)	0.779 (0.000, 8.248)	0.872	0.0 (0.0)	2.2 (2.1)	0.672 (0.000, 8.063)	0.801
Clavien-Dindo surgical complications ≥ 1	7 (25.0)	58 (36.7)	0.599 (0.215, 1.408)	0.268	5.5 (19.3)	39.7 (37.6)	0.423 (0.132, 1.069)	0.090
Postoperative hospital stay, days	12.5 (17.9)	11.1 (14.7)	1.449 (-4.664, 7.562)	0.642	10.90 (16.68)	10.15 (12.13)	0.750 (-4.813, 6.312)	0.792
In-hospital mortality	0 (0)	1 (0.6)	1.842 (0.000, 58.189)	0.714	0.0 (0.0)	1.0 (1.0)	1.208 (0.000, 32.219)	0.910

Values are mean (standard deviation) or number (%). Early vs. Late surgery: surgical time from coronary stent implantation within 6 months vs. after 6 months. IPTW, inverse probability treatment weighting; MACE, major adverse cardiovascular event; OR, odds ratio; CI, confidence interval. _a_ Logistic regression with Firth’s penalized maximum likelihood estimator (MLE). _b_ Weighted logistic regression.

**Table 3:** Multiple logistic regression for myocardial injury (raw data).

			Univariable Analysis		Multiple Logistic Regression	
	Myocardial Injury (n = 52)	No Myocardial Injury (n = 134)	OR, 95% CI	*p* Value	aOR, 95% CI	*p* Value
Early surgery	8 (15.4)	20 (14.9)	1.034 (0.418, 2.558)	0.937	1.249 (0.478, 3.262)	0.65
Age, year	70.0 (9.5)	67.4 (7.7)	1.045 (1.004, 1.089)	0.033		
BMI, kg/m^2^	24.9 (3.5)	24.6 (3.0)	1.032 (0.926, 1.150)	0.568		
Female	9 (17.3)	11 (8.2)	2.595 (0.964, 6.985)	0.059	2.749 (0.969, 7.795)	0.057
Surgery type	25 (48.1)	21 (15.7)	5.010 (2.399, 10.464)	<0.001	5.060 (2.407, 10.635)	<0.001
Major vascular surgery						
Emergency surgery	4 (7.7)	12 (9.0)	0.919 (0.274, 3.079)	0.891		
ASA PS						
II	21 (40.4)	60 (44.8)	(ref)			
III	30 (57.7)	71 (53.0)	1.100 (0.562, 2.154)	0.780		
IV	1 (1.9)	3 (2.2)	0.810 (0.080, 8.234)	0.858		
Etiology of stent insertion						
AMI	16 (31.4)	45 (37.2)	(ref)			
Angina pectoris	35 (68.6)	76 (62.8)	1.301 (0.646, 2.618)	0.461		
Unknown	1 (1.0)	13 (1.0)	NA			
DM	15 (28.8)	58 (43.3)	0.529 (0.262, 1.069)	0.076		
Metformin	5 (9.6)	28 (20.9)	0.403 (0.146, 1.108)	0.078		
Sulfonylurea	3 (5.8)	21 (15.7)	0.330 (0.094, 1.156)	0.083		
DPP-4-inhibitor	4 (7.7)	20 (14.9)	0.475 (0.154, 1.464)	0.195		
HTN	35 (67.3)	96 (71.6)	0.777 (0.383, 1.575)	0.483		
CKD	10 (19.2)	9 (6.7)	3.398 (1.190, 9.706)	0.022	2.583 (0.893, 7.468)	0.08
Atrial fibrillation	4 (7.7)	9 (6.7)	1.170 (0.336, 4.076)	0.805		
Peripheral vascular diseases	10 (19.2)	22 (16.4)	0.990 (0.418, 2.341)	0.981		
Any stroke or TIA	7 (13.5)	33 (24.6)	0.409 (0.158, 1.057)	0.065		
Preoperative antiplatelets						
None	1 (1.9)	2 (1.5)	(ref)			
Dual antiplatelets	26 (50.0)	61 (46.5)	<0.001 (0, Infinite)	0.981		
Single antiplatelet	25 (48.1)	71 (53.0)	<0.001 (0, Infinite)	0.986		
Discontinued days of antiplatelets	4 (1,5)	4 (1,5)	0.931 (0.839, 1.034)	0.182		

Values are presented as mean (standard deviation) or median (interquartile range), or number (%). Early vs. Late surgery: surgical time from coronary stent implantation within 6 months vs. after 6 months; OR odds ratio; aOR, adjusted odds ratio; CI, confidence interval; BMI, body mass index; ASA PS, American Society of Anesthesiologist Physical Status; AMI, acute myocardial infarction; DM, diabetes mellitus; DPP-4-inhibitor, dipeptidyl peptidase-4 inhibitor; HTN, hypertension; CKD, chronic kidney diseases; TIA, transient ischemic attacks.

## Data Availability

Not applicable.

## Appendix A

**Table A1:** Baseline Patient and Operative Characteristics after IPTW.

	Weighted Cohort after IPTW			
	Early Surgery (n = 28.3)	Late Surgery (n = 105.5)	*p*-Value	Test SMD
Age, year	66.91 (8.29)	68.11 (8.25)	0.533	0.145
Female	2.6 (9.3)	5.4 (5.1)	0.56	0.162
BMI, kg/m^2^	25.10 (2.68)	24.78 (3.18)	0.637	0.112
ASA PS ≥ IV	28.3 (100.0)	105.5 (100.0)	NA	<0.001
Nonvascular surgery	21.6 (76.3)	82.0 (77.7)	0.884	0.034
Emergency surgery	3.3 (11.6)	6.4 (6.1)	0.514	0.196
Etiology of stent insertion, angina pectoris	18.1 (63.7)	70.3 (66.6)	0.805	0.061
Use of any antiplatelets	28.3 (100.0)	105.5 (100.0)	NA	<0.001
Discontinued days of antiplatelets	4.97 (3.74)	3.70 (2.29)	0.214	0.409
*Comorbidity*				
Diabetus Melitus	11.4 (40.1)	40.9 (38.8)	0.917	0.026
Hypertension	22.3 (78.7)	71.0 (67.3)	0.26	0.258
Chronic kidney diseases	1.3 (4.5)	8.1 (7.6)	0.605	0.131
Preoperative atrial fibrillation	1.3 (4.5)	6.9 (6.5)	0.728	0.087
Peripheral vascular diseases	4.6 (16.4)	18.5 (17.5)	0.899	0.030
Stroke or TIA	4.4 (15.4)	18.9 (17.9)	0.775	0.068

Values are presented as mean (standard deviation) or number (%). Early vs. Late surgery: surgical time from coronary stent implantation within 6 months vs. after 6 months. IPTW, inverse probability treatment weighting; SMD, standardized mean difference; BMI, body mass index; ASA PS, American Society of Anesthesiologist Physical Status; TIA, transient ischemic event.
